# The impact of adolescent clinical depression and depressive symptoms on moral thinking: based on process dissociation approach

**DOI:** 10.3389/fpsyg.2025.1519595

**Published:** 2025-06-25

**Authors:** Mufan Zheng, Ziran Ma, Jin He

**Affiliations:** ^1^Department of Psychology, Wuhan University, Wuhan, China; ^2^Department of Psychiatry, The First Affiliated Hospital of Zhengzhou University, Zhengzhou, China

**Keywords:** moral thinking, process dissociation procedure, deontology, utilitarianism, clinical depression, subclinical depressive symptoms, adolescents, rumination

## Abstract

**Background:**

Adolescence is a critical period for moral development, and depression significantly impacts this process by altering cognitive and emotional processing, affecting the resolution of moral dilemmas. Rumination, closely linked to depression, also influences emotional and cognitive processing during moral judgments.

**Methods:**

Study 1 examined 34 depressed adolescents and 36 healthy controls who completed the Beck Depression Inventory-Short Form and Beck Anxiety Inventory, followed by 20 moral dilemmas from the Process Dissociation (PD) procedure. Study 2 (*n* = 568) explored subclinical depressive symptoms and their antecedent role of rumination on moral judgments. The SCL-90 scale measured depression, paranoid ideation, and hostility, while the Positive and Negative Rumination scales assessed rumination levels.

**Results:**

Clinically depressed adolescents showed significantly reduced reliance on both utilitarian [*t*(65) = −3.90, *p* < 0.001, Cohen’s *d* = 0.95, 95%CI(−0.18, −0.06)] and deontological tendencies [*t*(65) = −3.03, *p* = 0.004, Cohen’s *d* = 0.74, 95%CI (−0.25, −0.05)], compared to control group. Subclinical depressive symptoms predicted lower deontological tendencies [β = −0.13, *t*(566) = −3.09, *p* = 0.02, 95%CI (−0.05, −0.01)]. Sequential mediation analyses revealed: (a) Negative rumination → depression → paranoid ideation → deontological decline [Indirect effect: *b* = −0.003, 95%CI (−0.005, −0.001)]; (b) Negative rumination → depression → hostility → deontological decline [Indirect effect: *b* = −0.003, 95%CI (−0.005, −0.0004)]. Negative rumination exacerbated depressive symptoms, which sequentially increased paranoid ideation and hostility, ultimately lowering deontological judgments.

**Conclusion:**

Clinical depression decreases deontological and utilitarian moral reasoning, while negative rumination exacerbated depressive symptoms, which sequentially increased paranoid ideation and hostility, ultimately lowering deontological judgments. These findings highlight depression’s nuanced impact on adolescent moral development and underscore transdiagnostic mechanisms requiring targeted intervention.

## 1 Introduction

Depressive disorder, or depression, is a common mental health condition characterized by prolonged periods of low mood or a diminished interest in activities (World Health Organization [WHO], 2023). Adolescents are in a stage of growth and development, and the characteristics of adolescent depression differ from those of adults (Paus et al., 2008). Depression rates have risen sharply in adolescents compared to adults (Weinberger et al., 2018), raising concerns about its impact on social, emotional, and cognitive development during this critical period (Patton et al., 2016).

As a common mental illness, depression can significantly impact cognitive functions, including processes like moral judgment (Spear, 2000). Adolescents are in the process of forming moral concepts, often without stable moral values, and their stage of moral development also differs from that of adults (Koenig and Gao, 2022). Previous studies have studied how depression and depressive symptoms affect moral judgments (e.g., Laing et al., 2022; Yin et al., 2022), but most of them focus on adult depression, with less research on adolescent depression. Therefore, the impact of depression and depressive symptoms during adolescence on moral reasoning may exhibit characteristics that differ from those observed in adults. This study aims to explore the impact of adolescent depression and depressive symptoms on moral thinking.

When faced with moral dilemmas, people make choices and judgments about the rightness or wrongness of individual actions. These are known as moral judgments. The choice and implementation of different behaviors correspond to different psychological processes in people, which are essentially different moral judgments. Greene et al. (2001, 2004) proposed a dual-process model of moral judgment, where emotions and cognition compete to shape decisions. When emotions prevail, individuals tend to make deontological decisions, viewing harming as inherently wrong despite positive outcomes. Conversely, cognitive dominance leads to utilitarian decisions, prioritizing the greater good, even if harm is involved. For example, in the trolley problem (Thomson, 1986), diverting a runaway trolley to save five lives at the cost of one reflects utilitarian reasoning, whereas refusing to act reflects deontological principles. Consistent with dual-process model, When the ventromedial prefrontal cortex (vmPFC) associated with emotional experience is damaged, individuals tend to exhibit more utilitarian choices (Koenigs et al., 2007; Ciaramelli et al., 2007). In patients with frontotemporal dementia caused by alexithymia and depressive moods, an increase in utilitarian choices is also observed (Cecchetto et al., 2018; Laing et al., 2022).

Many researchers have conducted studies to discuss the relationship between depression and moral judgments. Laing et al. (2022) found that depression was associated with increased utilitarian responses in personal moral dilemmas involving emotionally-salient elements, while Andrade et al. (2023) found depressive symptoms predicted utilitarian choices in trolley dilemma. Gago et al. (2019) research showed increased utilitarian judgments in individuals with bipolar disorder due to reduced empathy and heightened focus on personal emotional states, like guilt. However, the studies mentioned above all rely on classical moral judgment paradigms, which position deontology and utilitarianism as opposing ends of a continuum, making it challenging to clearly differentiate between utilitarian and deontological tendencies (Conway and Gawronski, 2013).

Yin et al. (2022) employed process dissociation technology—which enables the distinct evaluation of utilitarian and deontological inclinations in moral judgments—to examine the relationship between depressive symptoms and these thinking tendencies. Their findings indicated that severe depressive symptoms are associated with a reduced inclination toward deontological judgment among subclinical adult populations. In the dual-process model (Greene, 2009), deontological tendencies are the result of emotional responses to harmful actions. The findings also indicate that this decrease in deontological judgment does not stem from a desire to optimize the wellbeing of the majority, but rather result from impaired cognitive control over moral judgments due to emotional interference. This aligns with findings that emotional deficits in depression hinder emotional processing in moral judgments (Cardinale et al., 2018; McCormick et al., 2016).

In brief, adult depression and depressive symptoms are usually associated with increased utilitarian judgments and decreased deontological tendency. However, previous studies have three major flaws that need to be resolved.

First, existing researches mainly indicated the impact of depression on moral judgments in adults, but adolescent studies remain scare. However, emerging evidence suggests that depressive symptoms uniquely disrupt moral reasoning in adolescents. For instance, adolescents with depressive moods exhibit altered social decision-making, such as increased acceptance of unfair proposals in gain-related contexts, potentially due to cognitive biases and social withdrawal (Hu et al., 2021). Furthermore, depression disrupts moral judgment by impairing value refinement processes; depressed adolescents demonstrate greater value deliberation but report reduced confidence in their choices, highlighting the complexity of depressive symptoms in moral contexts (Moses-Payne et al., 2024).

Adolescence is a critical phase for psychopathological development and marked by significant changes in emotional regulation and cognitive abilities (Casey et al., 2008; Crone and Dahl, 2012; Silvers et al., 2012; Steinberg, 2005). These developmental shifts may influence moral decision-making, as emotion and cognition are central to moral judgments according to the dual-process model (Greene, 2009). Research showed that emotions play a pivotal role in adolescents’ moral thinking processes: negative emotions, such as guilt, strongly predict moral choices in antisocial contexts, while positive emotions, such as pride, influence prosocial decisions (Krettenauer et al., 2011). Older adolescents increasingly prioritize outcome-oriented emotions, which may reduce the appeal of morally driven decisions (Krettenauer et al., 2013). These findings underscore the interplay between affective development and moral reasoning during adolescence.

There are also some circumstantial evidences that the characteristics of adolescents’ moral judgments differ from those of adults. Bacchini et al. (2021) found that interpersonal relationships and social contexts are pivotal in adolescents’ moral development, influencing their moral judgments. A study conducted in Italy (Koenig and Gao, 2022) found that, compared to adults, adolescents were more inclined to approve fewer personal forms of harm, highlighting the importance of peer attachment and emotional traits in shaping moral judgments. Another recent research conducted in Northern China by Jiang et al. (2025) reveals that adolescents exhibit a more utilitarian approach to trolley and footbridge moral dilemmas than adults, prioritizing outcomes that maximize overall welfare. This tendency persists across both individual and group decision-making contexts, underscoring developmental differences in moral reasoning between adolescents and adults (Jiang et al., 2025).

Although the findings of Koenig and Gao (2022) and Jiang et al. (2025) regarding the differences in moral judgment between adolescents and adults are not consistent, we believe this may be attributed to cultural differences. Nevertheless, both studies reveal that within their respective cultures, there are clear distinctions in moral thinking between adolescent and adult populations. This underscores the importance of studying adolescent moral judgment. As a unique stage of individual development, adolescents’ moral reasoning differs from that of adults, and therefore, findings from adult research cannot be directly applied to adolescents.

Second, previous studies primarily focused on non-clinical groups (e.g., Andrade et al., 2023; Laing et al., 2022; Yin et al., 2022) or bipolar disorders (Gago et al., 2019), leaving adolescent depression understudied. Major depressive disorder, as defined in the DSM-5, involves a depressed mood or loss of interest or pleasure lasting at least 2 weeks (American Psychiatric Association [APA], 2013). Meanwhile, the primary symptom of clinical depression must be accompanied by at least four additional symptoms, such as changes in sleep (insomnia or hypersomnia), appetite or weight fluctuations, difficulty concentrating, fatigue, psychomotor agitation or slowing, feelings of worthlessness or inappropriate guilt, and recurrent thoughts of death or suicide. In individuals under 18, irritability may also be present. Symptoms that do not meet the full criteria for major depressive disorder are classified as subclinical (subthreshold) depression (Cuijpers and Smit, 2008). Thus, it can be seen that there are differences between clinical depression and non-clinical depressive symptoms. In contrast, patients with clinical bipolar disorder exhibit alternating episodes of depressive and manic symptoms, which differ from the patterns observed in individuals with depression (Gago et al., 2019). Considering the current research gap, we aimed to investigate the impact of both clinical and subclinical depression on moral judgments in adolescents in the current study.

Third, there is no uniform conclusion on the mechanism of depression’s influence on moral judgments, and empirical research is lacking. Classical moral dilemmas treat deontology and utilitarianism as two bipolars in a continuum. The two are conflicting, and the growth of deontology means the decline of utilitarianism. Classic moral dilemmas make it difficult to distinguish between utilitarian and deontological inclinations (Conway and Gawronski, 2013), further obscuring the mechanism. Therefore, we followed Conway and Gawronski’s (2013) process dissociation paradigm to dissociate deontological and utilitarian tendencies to explore the influencing mechanism of depression on moral reasoning. Based on previous studies, depression has been found to promote utilitarian judgments (Laing et al., 2022; Gago et al., 2019; Andrade et al., 2023). Meanwhile, a study using process dissociation (Yin et al., 2022) found that depressive states reduce deontological decision-making specifically in adults. We hypothesized that adolescents with depression are more likely to rely on utilitarian inclinations and less likely to rely on deontological ones to make moral judgments compared to the control group.

Moreover, we aimed to explore the mechanism underlying the relationship between depression and moral judgments. According to the dual-process model (Greene, 2009), moral decision-making is influenced by the cognitive and emotional dual processing of individuals. Depression, whether as a clinical disorder or a subclinical symptom, affects moral judgment through some cognitive and emotional processes. We posited that the impact of clinical depression and subclinical depression on adolescents’ moral judgment may differ. While there is currently a lack of research on the influence of clinical depression on adolescents’ moral cognition, it is well-established that clinical depression, characterized by more severe symptoms and a longer course, affects neural activity, hormonal levels, cognition, and emotion (Beck, 2008). Consequently, adolescents with clinical depression may experience a deeper and more significant impact on their moral thinking compared to those who exhibit depressive symptoms but do not meet the criteria for clinical depression.

Therefore, in study 1, we specifically selected adolescents diagnosed with clinical depression who were hospitalized as participants, while in study 2, we recruited adolescents from regular school settings, to explore whether the varying degrees of depression exert different effects on adolescents’ moral cognition and moral judgment.

We selected two common cognitive and emotional mental symptoms as mediating variables, paranoid ideation and hostility. Paranoid ideation and hostility represent critical transdiagnostic dimensions that explain shared moral judgment patterns across comorbid conditions such as depression, borderline personality disorder, and anxiety disorders. Murphy et al. (2018) proposes that paranoia, including subclinical manifestations, functions as an adaptive defense mechanism to alleviate low self-esteem and depressive affect through hypervigilance toward social threats. This mechanism is transdiagnostic, prevalent in depression, anxiety disorders, and psychotic-spectrum conditions. Similarly, Beck (2008) identifies hostility—a core component of the “cognitive triad” involving negative beliefs about others—as a shared mechanism amplifying interpersonal threat perceptions in depression, bipolar disorder, and anxiety. These transdiagnostic features provide a foundation for extending the relationship between depression and moral decision-making to broader psychopathology.

Paranoia is defined as an unjustified distrust and suspiciousness toward others, with their motives often interpreted as malevolent (American Psychiatric Association [APA], 2013), which relates to a distorted cognitive process. Previous study showed that patients with paranoid schizophrenia tended to judge the actions of protagonists who attempted to harm another person more leniently compared to actions that were accidental, based on distorted interpretations of the other person’s intentions (Cyrkot et al., 2024). Similarly, Savulich et al. (2018) demonstrated that deficits in social cognitive processes associated with paranoid ideation contribute to maladaptive emotional responses to interpersonal harm, ultimately leading to alterations in prosocial behavior within economic exchange paradigms. Together, these findings suggest that the distorted interpretations inherent in paranoid thinking can drive decisions that deviate from normative moral judgments. There were also substantial evidence suggesting that individuals with depressive symptoms at both clinical and subclinical levels are more inclined to show paranoid ideation (e.g., Freeman et al., 2012; Grant et al., 2005; Kool et al., 2000; Martin and Penn, 2001). A longitudinal study also found that higher self-rated depressive symptoms were associated with more severe courses of paranoid ideation (Saarinen et al., 2018). Therefore, we expected that paranoia mediates the relationship between depression and moral judgments.

Hostility involves resentment, suspicion, and alienation (Berkowitz, 1993; Buss and Perry, 1992; Spielberger et al., 1983), often linked to aggression and interpersonal dysfunction. Individuals experiencing major depressive episodes exhibit heightened levels of hostility, which tend to intensify as the severity of depressive symptoms increases (Moreno et al., 1993). Additionally, feelings of hostility may promote the activation of moral disengagement mechanisms, enabling individuals to justify aggressive behaviors by reducing cognitive dissonance and circumventing moral self-sanctioning (Rubio-Garay et al., 2016). The moral disengagement allows individuals to disengage from moral standards, thus diminishing personal accountability for their actions (Caprara et al., 2009; De Caroli and Sagone, 2014). As a result, heightened moral disengagement may lead individuals to adopt more utilitarian approaches to moral judgment and may serve as a mediating factor in the relationship between depression and moral reasoning.

Also, previous studies showed depression is likely to lead to ignoring moral norm for the greater good, but no studies tried to discuss how to improve this problem. Emotion regulation refers to the process by which individuals influence the emotions they experience, when they experience them, and how they are expressed (Gross, 1998; Gross, 2015). Gross (1998) proposed the Emotion Regulation Process Model and growing studies show that emotion regulation could significantly help to improve the symptoms of mental illness across various stages of life (Aldao et al., 2016; Chu et al., 2017; Cludius et al., 2020; Essau et al., 2017).

Emotion regulation is a key transdiagnostic factor in many psychiatric disorders including depression (Aldao et al., 2010; Sheppes et al., 2015; Sloan et al., 2017). Individual differences in emotion regulation, particularly impaired emotion regulation ability, are linked to the risk of developing various psychopathologies. Maladaptive emotion regulation strategies like suppression and avoidance have been shown to mediate the relationship between emotional distress and mental health issues in both children and adults (Andrés et al., 2016; Yoon et al., 2013). Socially, youth with difficulties in emotion regulation often struggle with social competence (Fabes and Eisenberg, 1992), face challenges forming close relationships (Yap et al., 2007), and may experience social isolation and increased negative affect, which contribute to mental health problems (Yap et al., 2007). The current study explores rumination—a common emotion regulation strategy (Liu and Thompson, 2017)—as a potential mechanism to examine how depression influences moral judgments.

Rumination, an emotion regulation strategy, involves repetitively focusing on emotional experiences, their causes, and consequences without actively seeking solutions (Nolen-Hoeksema et al., 2008). There is some longitudinal research showing that rumination strongly associated with internalizing psychopathologies, particularly depression and anxiety, in both adults and youth (McLaughlin and Nolen-Hoeksema, 2011; Rood et al., 2009; McLaughlin et al., 2011; Nolen-Hoeksema et al., 2007). Also, excessive rumination has been found to mediate the connection between childhood maltreatment and the development of generalized psychopathology up to 2 years later (Weissman et al., 2019).

Negative rumination has been shown to reinforce depressive symptoms (Aldao et al., 2010; Nolen-Hoeksema, 2000) and to promote utilitarian moral judgments through disruptions in cognitive-emotional processing (Stuhrmann et al., 2011). However, rumination is not exclusively maladaptive; positive rumination, characterized by reflective focus on positive attributes and experiences to enhance emotional states, has also been identified (Larsen and Prizmic, 2008; Feldman et al., 2008). Similar to negative rumination, positive rumination can occur automatically and may develop into a habitual, trait-like cognitive style, with some individuals demonstrating a stable tendency toward positive rumination (Watkins and Nolen-Hoeksema, 2014; Yang and Guan, 2022). While negative rumination typically exacerbates negative affect and increases vulnerability to psychopathology, positive rumination has been found to elevate positive mood and is inversely associated with depressive symptoms both cross-sectionally (Feldman et al., 2008) and prospectively (Nelis et al., 2016). Accordingly, positive rumination may serve as a protective factor against psychological difficulties such as depression and anxiety (Naragon-Gainey et al., 2009). Based on this, it can be hypothesized that positive rumination may contribute to a reduction in depressive symptoms, which in turn could lead to a decrease in utilitarian moral judgments.

In brief, we hypothesized that negative rumination will increase depressive symptoms, subsequently leading to higher utilitarian thinking and lower deontological thinking through increased paranoia and hostility. In opposite, the use of positive rumination will reduce depression and subsequently diminish its impact on moral judgments. In summary, we hypothesized the serial mediation effects of “negative rumination n effects of d paranoia and hostility. In judgments” and “negative rumination n effects of d paranoia and hostijudgments.”

To address the identified three issues mentioned above, we conducted two studies. In Study 1, we examined the impact of clinical depression on moral judgments, using process dissociation to differentiate between utilitarian and deontological tendencies. Study 2 extended the investigation to adolescents with subclinical depression, examining rumination as an antecedent variable and paranoid ideation and hostility as mediating mechanisms, in order to further elucidate the relationship between depressive symptoms and moral judgments.

## 2 Study 1

Study 1 explores whether adolescents with clinical depression differ in moral judgments compared to a control group. Using the process dissociation method, it investigates the impact of depression on utilitarian and deontological inclinations.

### 2.1 Methods

#### 2.1.1 Participants

Prior to data collection, we conducted a power analysis using G*Power based on an independent samples *t*-test. We set the parameters as follows: Cohen’s *d* = 0.7 (indicating a medium-to-large effect size), power = 0.80, and α = 0.05. The analysis indicated that at least 34 participants per group (a total of 68) were required. Our final sample met these criteria. The participants, all aged between 12–20 years, were 34 adolescents with depression (*M* = 16.97, *SD* = 0.53) from the Psychiatry Department at The First Affiliated Hospital of Zhengzhou University (Zhengzhou, China) and 33 healthy adolescents (*M* = 15.24, *SD* = 0.24) recruited through Junior high school (Qisheng School, Shantou, China).

The clinical participants were initially assessed in outpatient clinics or hospital wards, where psychiatrists conducted structured diagnostic interviews using the Mini-International Neuropsychiatric Interview (MINI), a brief structured interview aligned with ICD-10 criteria for depressive disorders. The MINI systematically evaluates key depressive symptoms—such as persistent low mood, loss of interest or pleasure, changes in appetite and sleep, and various cognitive and somatic symptoms—to ensure that each patient met the diagnostic standards outlined by the ICD-10 (World Health Organization [WHO], 1992). Following this, diagnoses were confirmed via case note reviews and further verified by the responsible psychiatrist. Additionally, a clinical expert in our research team conducted an evaluation using the Hamilton Depression Scale (HAMD) to corroborate the diagnosis.

Exclusion criteria for patients included: a history of neurological conditions or major medical disorders that could impact cognition; use of non-psychotropic medications that might affect cognition (e.g., corticosteroid treatment); and other psychiatric diagnoses according to ICD-10 criteria (e.g., comorbidity anxiety) (World Health Organization [WHO], 1992).

Because we aimed to ensure that participants in the control group did not have depression or any other mental or physical illnesses that could be associated with depression, we recruited control group participants from schools. Healthy controls were recruited from schools without prior screening in an effort to boost ecological validity. Subsequently, during *post hoc* evaluation, those who potentially surpassed the clinical depression thresholds (BDI—13 > 5 and PHQ—9 > 5) or had a history of any psychiatric conditions were excluded. A total of 23 participants were excluded from the original sample (39 patients, 47 healthy controls) on the basis of these criteria.

#### 2.1.2 Measures and procedure

The Hamilton Depression Scale. The Hamilton Depression Scale (HAMD-24) is an essential tool for measuring depression. The Hamilton Rating Scale for Depression was developed by Hamilton (1960) and remains a widely used clinical assessment. The HAMD consists of a 24-item scale, each item scored on a severity scale from 0 to 4, with some items scored from 0 to 2. In this study, the scale is used for secondary confirmation of the diagnosis of clinical depression in patients.

Patient Health Questionnaire (PHQ-9). The Chinese version of the PHQ-9 (Zhou et al., 2021) is used to measure depression. The total score ranges from 0 to 27, summing the scores of its nine items, each rated on a 4-point Likert scale from 0 (not at all) to 3 (nearly every day). This scale assesses the severity of depressive episodes in the general population and is recognized for its strong psychometric properties, including high internal consistency. In the present study, the Cronbach’s α was 0.88.

Beck Depression Inventory 13-item Version (BDI-13). This shorter version of the Beck Depression Inventory is widely used and correlates highly (*r* = 0.96) with the original version and clinical ratings of depression severity (Beck and Beck, 1972). Bennett et al. (1997) reported similar diagnostic efficiency for both versions. The BDI-13 contains 13 items, each scored from 0 to 3, with total scores ranging from 0 to 39. In the present study, the Cronbach’s α was 0.90.

Beck Anxiety Inventory (BAI). The Beck Anxiety Inventory (BAI), developed by Beck et al. (1988), consists of 21 items related to anxiety symptoms. Participants rate each item from 0 to 3 based on their experiences over the past week. The total score is calculated by summing the scores of all 21 items, ranging from 0 to 63, with higher total scores indicating greater severity of anxiety in the participant. In the present study, the Cronbach’s α was 0.95.

Participants in the experimental group were tested individually in a quiet room. After signing informed consent forms, participants responded to demographic and clinical interviews as well as clinical scales, which include BDI-13, PHQ-9, BAI. Subsequently, they completed the moral judgments tasks. The control group was recruited in a middle school. Students were invited to voluntarily complete the clinical scales and moral judgment tasks during the class.

After participants completed the questionnaires, the experimenters carefully reviewed each response to ensure that no items were left unanswered; therefore, there were no issues with missing data.

#### 2.1.3 Moral dilemmas

Using the process dissociation (PD) procedure allows for the separate assessment of the inclination of utilitarian and deontological inclinations in moral judgments. This analytical separation is achieved through the paradigm’s core mechanism: the systematic deployment of congruent and incongruent moral dilemmas (Conway and Gawronski, 2013). By contrasting responses across these two dilemma types, the PD procedure effectively circumvents the conceptual overlap inherent in traditional approaches that force dichotomous categorization along a single continuum. In implementation, participants engaged with 10 of each type, sourced from Conway and Gawronski (2013).

Incongruent dilemmas echo classic high-conflict situations where the morally contentious act leads to a better overall outcome. These dilemmas elicit conflicting responses based on the participant’s utilitarian or deontological tendencies. For instance, the classical scenario involves a crying infant where participants weigh the option of smothering the baby to save a group from being killed. Here, a utilitarian inclination would justify the action for the greater good, whereas a deontological stance would reject it due to the inherent wrongness of the act. Congruent dilemmas, while structurally similar to their incongruent counterparts, differ in their outcomes—harmful actions result in more unfavorable consequences (Conway and Gawronski, 2013). Consequently, both utilitarian and deontological tendencies would converge toward a common response in these scenarios. For example, in the congruent version of the crying baby scenario, participants had to consider if it was appropriate to smother the child to avoid others being captured for forced labor in the quarries, where the harm does not prevent a greater evil but leads to a negative outcome, both inclinations would typically oppose the action. The examples of dilemmas, crying baby and vaccine policy, can be found in Appendix.

**FIGURE 1:**
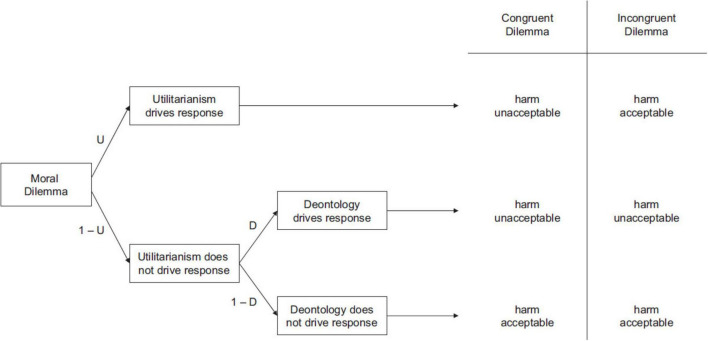
Processing tree illustrating the underlying components leading to judgments that harmful action is either acceptable or unacceptable in congruent and incongruent moral dilemmas. The paths from left to right depict the three cases that **(a)** utilitarianism ultimately drives the response, **(b)** deontology ultimately drives the response, and **(c)** neither utilitarianism nor deontology drives the response (Conway and Gawronski, 2013).

Participants’ judgments in congruent and incongruent dilemmas can be further clarified by means of a processing tree (see Figure 1) under the Process Dissociation (PD) framework (Conway and Gawronski, 2013). Each path from left to right in the processing tree represents different judgment outcomes on these two types of dilemmas, depending on which psychological process ultimately drives the response: (a) Utilitarianism (U) drives the response (top path), (b) Deontology (D) drives the response (middle path), (c) Neither utilitarianism (1 – U) nor deontology (1 – D) drives the response (bottom path). In congruent dilemmas, harm is judged unacceptable if utilitarianism drives the response (U) or if deontology drives the response (D) when utilitarianism does not (1 – U). Harm is only deemed acceptable in congruent dilemmas when neither utilitarianism nor deontology influences the judgment (1 – U and 1 – D). In incongruent dilemmas, however, harm is judged acceptable when utilitarianism drives the response (U) or when neither utilitarianism nor deontology is in play (1 – U and 1 – D). Conversely, it is judged unacceptable when deontology drives the response (D) in the absence of a utilitarian driver (1 – U). By measuring the frequency of “harm-unacceptable” (or “harm-acceptable”) decisions across various moral scenarios, the PD procedure allows researchers to disentangle and quantify distinct utilitarian and deontological tendencies in moral judgment.

In the PD procedure, Utilitarianism (U) is calculated by the formula:


U=p⁢(unacceptable/congruent)-p⁢(unacceptable/incongruent).

Scores of U range from −1 to 1.

Deontology (D) is calculated by the formula:


D=p⁢(unacceptable/congruent)/(1-U).

Scores of D range from 0 to 1.

A high score in U indicates participants tend to accept harmful actions when they could maximize good outcomes, whereas a high score in D indicates an aversion to harmful behaviors regardless of whether doing so maximizes consequences or not.

The PD approach has been widely used to study moral cognition and moral judgment in different populations (e.g., Conway and Gawronski, 2013; Yin et al., 2022). Although Process Dissociation (PD) procedure has not previously been applied to the study of moral judgment in adolescents, the moral dilemmas used in the PD paradigm are either classic moral dilemmas (e.g., crying baby) or well-established variants of them (Conway and Gawronski, 2013). Prior research has employed these classic moral dilemmas to investigate adolescents’ moral judgment and moral reasoning (e.g., Bacchini et al., 2021; Jiang et al., 2025; Koenig and Gao, 2022). These studies have consistently demonstrated that adolescents are capable of understanding such scenarios and making judgments based on their own thoughts. Therefore, we consider the PD procedure to be a valid and appropriate method for studying moral cognition in adolescent populations.

### 2.2 Results

Means, standard deviations, and correlations among the primary study variables are presented in Table 1. Independent samples *t*-tests revealed significant differences between depression patients and the control group in their inclinations toward utilitarianism and deontology judgments. Specifically, depression group showed lower utilitarian tendency (*M* = 0.04, *SD* = 0.12) compared to the control group (*M* = 0.16, *SD* = 0.13), *t* (65) = −3.90, *p* < 0.001, Cohen’s *d* = 0.95, 95%CI (−0.18, −0.06). Depression group showed lower deontological inclinations (*M* = 0.45, *SD* = 0.22) than the control group (*M* = 0.60, *SD* = 0.19), *t* (65) = −3.03, *p* = 0.004, Cohen’s *d* = 0.74, 95%CI (−0.25, −0.05).

**TABLE 1:** Means, standard deviations, and *t*-test comparisons among the variables in study 1.

	Control (*n* = 33) Mean (SD)/n (%)	Depression patient (*n* = 34) Mean (SD)/n (%)	*t*	Cohen’s *d*	95%CI
% Female	36	68			
Age	15.24 (0.24)	16.97 (0.53)			
BDI 13-item	2.27 (0.43)	20.41 (1.39)	−12.31***	−3.03	[15.20, 21.08]
PHQ-9	1.97 (0.33)	15.44 (1.05)	−12.06***	−2.96	[11.24, 15.70]
BAI	2.42 (0.40)	7.65 (2.79)	−1.82	−0.45	[−0.50, 10.94]
PD Utilitarianism	0.16 (0.02)	0.04 (0.02)	−3.90***	0.95	[−0.18, −0.06]
PD Deontology	0.60 (0.03)	0.45 (0.03)	−3.03**	0.74	[−0.25, −0.05]

**p < 0.01, ***p < 0.001.

Also, we conducted ANOVA analyses with controlling for age and gender, and found that depression group showed lower utilitarian tendency compared to the control group, *F* (1, 63) = 9.30, *p* = 0.003, η*_*p*_^2^* = 0.13, and depression group showed lower deontological approach than the control group, *F* (1, 63) = 14.03, *p* < 0.001, η*_*p*_^2^* = 0.18.

### 2.3 Discussion

The aim of Study 1 was to examine differences in moral judgments between adolescents with clinical depression and a healthy control group. Independent samples *t*-tests revealed that both utilitarian and deontological judgments were lower in the depressed group compared to the control group. According to Conway and Gawronski (2013), deontological tendencies are rooted in emotional reactions to harmful actions, while utilitarian tendencies are related to individual differences in cognitive demand. These findings indicate that both cognitive and emotional processes involved in moral judgments were affected by depression.

In Study 1, the participants’ ages ranged from 12 to 20, which covers a relatively wide age span. Given that adolescents undergo significant developmental changes across different ages, we included age as a control variable in our data analysis, and we found that after controlling for age, the impact of clinical depression on moral cognition remained unchanged. Additionally, because we conducted a sample size calculation using G*Power prior to data collection and adhered to this sample size, we believe the findings of the study are reliable. Nevertheless, we acknowledge that validating these findings with a larger sample would yield more robust results. Therefore, in Study 2, we narrowed the age range to 12–16 years and increased the sample size to enhance the reliability of the findings.

## 3 Study 2

Study 2 examines the effects of depressive symptoms on moral judgments in community adolescents and explores the impact of positive and negative rumination as antecedents. It also investigates the serial mediation effects of “negative rumination → depression → hostility → moral judgments” and “negative rumination → depression → paranoid ideation → moral judgments.”

### 3.1 Methods

#### 3.1.1 Participants

We collected 568 valid questionnaires from seventh and eighth grade students (age range from 12 to 16 years) at Zhengzhou No. 60 Middle School, China. Questionnaires were excluded if they (a) had incomplete sections on the moral dilemma tasks or depression scales, or (b) failed the validation questions (e.g., inconsistent responses to repeated items).

#### 3.1.2 Measures and procedure

The Symptom Checklist-90 (SCL-90). The SCL-90 (Derogatis and Cleary, 1977) is a self-reporting inventory consisting of 90 items. It covers nine symptom dimensions: somatization, obsessive-compulsive symptoms, interpersonal sensitivity, depression, anxiety, hostility, phobic anxiety, paranoid ideation, and psychoticism. Additionally, there are seven items that assess sleep and eating disturbances, which are part of the additional items. The inventory uses a five-point Likert scale from 0 (“not at all”) to 4 (“extremely”) to rate symptoms. The General Symptomatic Index (GSI) is calculated by dividing the total score by 90. The scores for each factor are calculated by dividing the total score of the items that make up a factor by the number of items that constitute that factor. The SCL-90 is designed to be a multidimensional measure for assessing a broad range of psychological problems and symptoms of psychopathology. It is widely used in both clinical and non-clinical populations and has been validated for use in various cultural backgrounds. Also, this scale has been validated in the adolescent populations (Rytilä-Manninen et al., 2016). In the present study, the Cronbach’s α of SCL-90 was 0.98.

The Positive and Negative Rumination Scale. The Positive and Negative Rumination Scale (PANRS), developed by Yang et al. (2020), measures individuals’ positive or negative ruminative thoughts toward positive and negative emotions (Yang et al., 2020). The scale consists of 23 items, with 10 items measuring positive rumination and 13 items measuring negative rumination. Items are scored on a 4-point Likert scale, ranging from 1 (“never”) to 4 (“always”), and the total score for each subscale is calculated by summing the scores, with higher scores indicating a higher level of ruminative thinking. It includes two second-order factors: Positive and Negative Rumination, as well as five first-order factors: Enjoyment of Pleasure, Suppression of Pleasure, Active Coping, Self-Doubt, and Negative Attribution. This questionnaire has been previously validated in adolescent populations and has been shown to be effective. Cronbach’s α of positive rumination was 0.81 while Cronbach’s α of negative rumination was 0.85 (Yang et al., 2020). In the present study, Cronbach’s α of positive rumination was 0.85 while Cronbach’s α of negative rumination was 0.86.

Researchers distributed paper questionnaires during self-study classes, instructing students to complete them according to the given instructions. Participants filled in demographic variables, the PANRS, SCL-90, and moral judgment tasks (the same as in Study 1). The questionnaires were collected uniformly after the class.

Because study 2 used self-report tools, we took some steps to control for social desirability and common method bias. Firstly, participants were informed that their responses would be anonymous and confidential, which is intended to reduce social desirability bias by alleviating concerns about judgment or repercussions related to their answers. Second, we balanced question design. We incorporated reverse-coded items and balanced questions across different scales to reduce the tendency to respond in a socially desirable manner and to encourage more accurate self-reflection. This also helps to counteract common method bias by making it more difficult for participants to maintain consistent response patterns that are driven by response styles rather than their true experiences. Additionally, participants were explicitly instructed to answer as honestly as possible and to reflect on their true experiences. This was emphasized at the start of the survey and in follow-up reminders to encourage genuine responses. Attention-check items were also adopted to exclude careless responders.

#### 3.1.3 Data analysis

The Statistical Analyses were conducted using SPSS (version 26). Harman’s single- factor test was used to test for common method bias (Podsakoff et al., 2003). The mediation model was investigated using the PROCESS macro developed by Hayes (2018).

If a participant had missing data on either the depression scale or the PD paradigm questionnaire, their entire response set was excluded from the analysis. However, if missing data occurred on other scales, we retained the participant’s data but excluded the specific scales with missing responses from the corresponding analyses.

### 3.2 Results

The data for this study were collected through self-report measures, posing a risk for common method bias. To assess this, we used Harman’s single-factor test. Exploratory factor analysis without rotation revealed 14 eigenvalues greater than 1, with the first factor accounting for 33.364% of the total variance, below the 40% threshold for substantial bias. These findings suggest that common method bias is not significant in this study.

#### 3.2.1 Descriptive statistics and correlation analysis

The descriptive statistics and correlation analysis are presented in Table 2.

**TABLE 2:** Descriptive statistics and correlation analysis of SCL-90 and its factors, positive and negative rumination, PD deontology, and PD utilitarianism in study 2.

	Mean	*SD*	*N*	1	2	3	4	5	6	7	8	9	10
1. Sex	0.52	0.50	538	1									
2. Age	13.97	0.76	538	−0.11**	1								
3. SCL-90 GSI	0.80	0.70	554	0.22***	0.05	1							
4. Depression	0.81	0.78	554	0.22***	0.07	0.94***	1						
5. Hostility	0.92	0.95	554	0.18***	−0.03	0.85***	0.78***	1					
6. Paranoid ideation	0.74	0.78	554	0.17***	0.03	0.88***	0.81***	0.77***	1				
7. Positive rumination	23.86	5.89	564	−0.06	0.09*	−0.28***	−0.28***	−0.26***	−0.21***	1			
8. Negative rumination	25.27	7.15	564	0.14**	0.12**	0.75***	0.73***	0.59***	0.67***	−0.13**	1		
9. Utilitarianism (U)	0.09	0.15	568	−0.03	−0.008	−0.02	−0.05	−0.003	−0.006	0.07	−0.02	1	
10. Deontology (D)	0.51	0.20	568	−0.05	−0.08	−0.14**	−0.13**	−0.16***	−0.19***	0.03	−0.12**	−0.14**	1

*p < 0.05, **p < 0.01, ***p < 0.001.

#### 3.2.2 Regression analysis

We conducted regression analyses on the relationships between depression and moral judgments, as well as positive and negative rumination and moral judgments. Depression was negatively correlated with deontological tendencies in moral judgments, β = −0.13, *t*(566) = −3.09, *p* = 0.02, 95%CI (−0.05, −0.01). However, there was no correlation between depression levels and utilitarian tendencies in moral judgments, β = −0.05, *t*(566) = −1.17, *p* = 0.24, 95%CI (−0.03, 0.007). After controlling for age and gender, depression was still negatively related to deontological tendencies, β = −0.11, *t*(566) = −2.45, *p* = 0.015, 95%CI (−0.05, −0.005), and has no correlation with utilitarian tendencies, β = −0.06, *t*(566) = −1.38, *p* = 0.17, 95%CI (−0.03, 0.005).

Depression was significantly negatively correlated with positive rumination, β = −0.28, *t*(566) = −6.82, *p* < 0.001, 95%CI (−2.69, −1.49), and significantly positively correlated with negative rumination, β = 0.73, *t*(566) = 25.34, *p* < 0.001, 95%CI (6.21, 7.25). Negative rumination was negatively correlated with deontological tendencies, β = −0.12, *t* (566) = −2.88, *p* = 0.004, 95%CI (−0.006, −0.001). However, positive rumination was not correlated with either the deontological (D) or utilitarian (U) parameters of moral judgments (*ps.* > 0.05).

#### 3.2.3 Mediation analysis

We conducted further regression analysis using Model 6 of the SPSS macro PROCESS to test the mediation pathways: “negative rumination → depression → paranoid ideation → deontological tendency” and “negative rumination → depression → hostility → deontological tendency.” Negative rumination was the independent variable (X), deontological tendency the dependent variable (Y), depression the first mediator (M1), and paranoid ideation or hostility the second mediator (M2) (see Table 3). Sex and age were controlled for.

**TABLE 3:** Serial mediation models of negative rumination→ depression → paranoid ideation → deontological thinking and negative rumination→ depression → hostility → deontological thinking in study 2.

Variables															
	*M1*			Model 1: M2 = Paranoid ideation			Y			Model 2: M2 = Hostility			*Y*		
	β	*SE*	*t*	β	*SE*	*t*	β	*SE*	*t*	β	*SE*	*t*	β	*SE*	*t*
Age	0.003	0.03	0.1	−0.04	0.03	−1.47	−0.02	0.01	−2.20*	−0.10	0.03	−3.01**	−0.03	0.01	−2.29*
Sex	0.18	0.05	3.72***	−0.01	0.04	−0.31	−0.01	0.02	−0.60	−0.003	0.05	−0.06	−0.01	0.02	−0.56
*X*	0.08	0.003	23.72***	0.02	0.004	4.29***	−0.0003	0.002	−0.19	0.005	0.005	0.96	−0.001	0.002	−0.70
*M1*				0.7	0.04	18.48***	0.03	0.02	1.23	0.93	0.05	19.15***	0.02	0.02	0.77
*M2*							−0.06	0.02	−3.25**				−0.04	0.01	−2.46*
*R2*	0.55			0.67			0.04			0.63			0.03		
*F*	208.77***			259.69***			4.60***			216.58***			3.67**		

(*X*) Negative rumination, (*M1*) Depression, (*Y*) Deontological thinking. *p < 0.05, **p < 0.01, ***p < 0.001.

**FIGURE 2:**
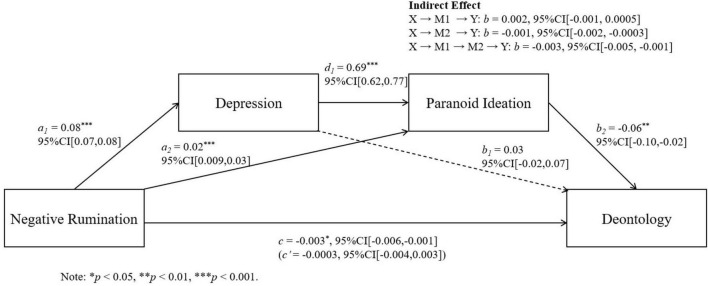
Serial mediation models of negative rumination, depression, and paranoid ideation on deontological judgments.

**FIGURE 3:**
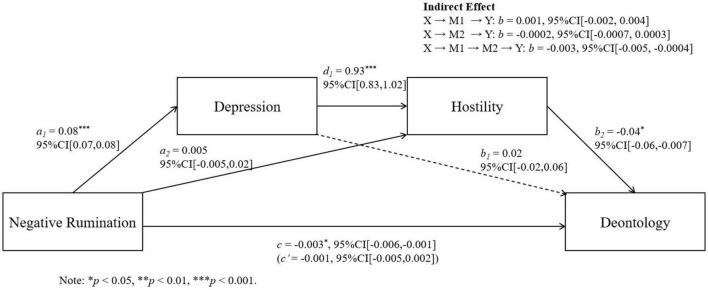
Serial mediation models of negative rumination, depression, and hostility on deontological judgments.

Results showed that both mediation pathways were significant (Table 4). The serial mediation models are illustrated in Figures 2, 3.

**TABLE 4:** Effects of pathways in serial mediation models of negative rumination→ depression → paranoid ideation → deontological thinking and negative rumination→ depression → hostility → deontological thinking in study 2.

Path			*M2* (Paranoid ideation)			*M2* (Hostility)	
		Effect	Se	95%CI	Effect	Se	95%CI
*c*	Total effect	−0.003	0.001	[−0.005, −0.0003]	−0.003	0.001	[−0.005, −0.0003]
*c*’	Direct effect	−0.000	0.002	[−0.004, 0.003]	−0.001	0.002	[−0.005, 0.002]
	Total indirect effect	−0.002	0.001	[−0.005, 0.000]	−0.002	0.001	[−0.004, 0.001]
*a1b1*	*X*→ *M1*→ *Y*	0.002	0.001	[−0.001, 0.005]	0.001	0.002	[−0.002, 0.004]
*a2b2*	*X*→ *M2*→ *Y*	−0.001	0.0004	[−0.002, −0.0003]	−0.0002	0.0002	[−0.0007, 0.0003]
*a1d1b2*	*X*→ *M1*→ *M2*→ *Y*	−0.003	0.001	[−0.005, −0.001]	−0.003	0.001	[−0.005, −0.0004]

### 3.3 Discussion

Study 2 aimed to extend the findings of Study 1 by shifting the focus from clinically depression to subclinical depressive symptoms among adolescents. The findings suggest that adolescents with higher levels of depressive symptoms demonstrated fewer deontological inclinations, while no difference was found in utilitarian inclinations. Also, we found that negative rumination influences deontological tendencies through two serial mediation pathways: “negative rumination → depression → hostility → moral judgments” and “negative rumination → depression → paranoid ideation → moral judgments.”

## 4 General discussion

This article, across two studies, employs the process dissociation method to differentiate between utilitarian and deontological tendencies, investigating the impact of clinical depression and subclinical depressive symptoms on moral judgments in adolescents. We also examined rumination as the antecedent, and the potential mediating role of paranoid ideation and hostility.

Study 1 revealed that adolescents with clinical depression exhibited reduced tendencies in both deontological and utilitarian moral judgments, and after controlling age and gender, this effect remained significant. Previous clinical studies have primarily concentrated on patients diagnosed with bipolar disorder. These studies have revealed that individuals with bipolar disorder tend to make utilitarian choices more frequently than healthy individuals when experiencing manic or depressive episodes (Gago et al., 2019; Kim et al., 2015). Furthermore, this tendency has also been observed in individuals in euthymic states (Larsen et al., 2019). Also, research shows that alcohol dependence patients have a higher inclination for utilitarian judgments in personal dilemmas, with this tendency being more pronounced in those with mild depression (Khemiri et al., 2012). However, rare research has studied clinical depression patients’ reason in moral dilemmas, especially adolescent patients. Adolescence is a crucial period for moral development, and the moral thinking of adolescents differs significantly from that of adults (Koenig and Gao, 2022). Depression may significantly impact adolescent moral development (Flouri and Ioakeimidi, 2018; Porter-Vignola et al., 2022), especially given the high prevalence (Birmaher et al., 1996).

Meanwhile, these studies adopted the traditional dilemma paradigm to assess participants’ moral choices, and did not differentiate between utilitarian and deontological approaches, potentially conflating the results to show an increase in utilitarianism. Nevertheless, our study used the process dissociation method to test moral thinking tendencies in a clinical group, which helps to clarify the influence of depression on deontological and utilitarian thinking respectively.

In Study 1, we discovered that patients with clinical depression exhibited lower tendencies toward both deontological and utilitarian moral thinking, consistent with our hypothesis. Previous studies mainly focused on adult patients with bipolar disorder in the depressive phase, and found that they have a higher tendency to rely on utilitarianism to make moral judgments compared to the control group (Gago et al., 2019; Kim et al., 2015). Our findings are different from theirs. There might be two reasons. Firstly, their studies did not separate utilitarian and deontological tendencies, but treat them as two bipolars in a continuum. Therefore, we show the impact of clinical depression on both deontological and utilitarian tendencies.

Also, neurobiological evidence suggests potential age-related differences in moral decision-making mechanisms. A comparative meta-analysis by Tang et al. (2018) revealed distinct amygdala-centered network dysfunctions in adults and adolescents with major depressive disorder. Adolescent patients showed impaired functional connectivity in the affective network and cognitive control network (e.g., reduced amygdala-dorsolateral prefrontal cortex connectivity). This suggested a reduction in both emotional network and the cognitive control network, which also explains why adolescents with clinical depression simultaneously exhibit both decreased utilitarian and decreased deontological tendencies. In contrast, adults exhibited abnormalities primarily within the affective network (e.g., heightened amygdala-hippocampal connectivity), potentially influencing emotional salience attribution without directly impairing utilitarian reasoning. This developmental divergence could explain why adolescents with depression demonstrate both lower utilitarian and deontological tendencies in our study, whereas adult patients in prior work showed different patterns.

According to Conway and Gawronski (2013), this simultaneous decline in both utilitarian and deontological tendencies observed in adolescents with clinical depression may suggest that they place less emphasis on both moral principles, and that other factors beyond these two frameworks may influence their moral judgments. This may also indicate a lack of concern for moral issues, which warrants further investigation in future research.

In Study 2, we aimed to study the impact of subclinical depressive symptoms on moral thinking. Different from Study 1, Study 2 found the negative correlation between subclinical depressive symptoms and deontological tendencies, but no relationship between depressive symptoms and utilitarian tendencies. Andrade et al. (2023) and Laing et al. (2022) found that higher levels of depressive symptoms in healthy adults were associated with a greater tendency toward utilitarian judgments. Taking this further, Yin et al. (2022) utilized process dissociation and found that higher levels of depressive symptoms correlated with fewer deontological judgments rather than an increase in utilitarian judgments. Our findings are consistent with the results of Yin et al. (2022).

According to the dual process model (Greene, 2009), deontological moral judgments are rooted in emotional reactions to harmful actions, while utilitarian judgments are driven by cognitive process. Higher depressive symptoms in the subclinical group led to a decrease in deontological tendencies, suggesting that the main impact of depression on moral judgments is rooted in emotional reactions.

However, our Study 1 found that the utilitarian tendencies of patients with depression also decreased, which reveals that adolescent clinical depression affects not only emotional responses but also cognition. This suggests that the influences of clinical depression and subclinical depressive symptoms on moral thinking are different. This could be due to the fact that in healthy populations with subclinical depressive symptoms, their depressive symptoms may be milder than those of clinical depression patients, possibly only affecting their emotions and not cognitive functions. For adolescent patients diagnosed with clinical depression, their symptoms are more severe, potentially already causing a greater impact on cognition. As demonstrated by Tang et al. (2018), adolescents with major depressive disorder exhibit hypoactive functional connectivity between the amygdala and the dorsolateral prefrontal cortex (DLPFC)—a core node of the cognitive control network (CCN)—which directly undermines executive functioning and goal-directed decision-making. Cognitive issues in individuals with major depressive disorder have been reported to occur in 85–94% of cases during depressive episodes and in 39–44% of cases during periods of remission (Conradi et al., 2011). It is recognized that cognitive disorders are a fundamental aspect of the clinical presentation of depression (Miskowiak et al., 2016). Furthermore, subclinical depression is more prevalent and intricate. While it is a risk factor for clinical depression (Cuijpers and Smit, 2004, Kessler et al., 1997), it can also manifest in anxiety disorders, bipolar disorder, schizophrenia, and other psychiatric disorders. This gives rise to a multitude of complex interactions.

Regarding the mediation analysis, we examined the antecedent role of negative rumination. Although both positive and negative rumination are correlating with depression, we only found that negative rumination is associated with fewer deontological judgments. Through mediation analysis, we discovered that negative rumination can exacerbate depressive symptoms, leading to increased paranoid ideation and consequently fewer deontological decisions. Depression leads to higher levels of paranoid ideation (Freeman et al., 2012; Grant et al., 2005; Kool et al., 2000), which causes biases in understanding and judging the harmful intentions in moral situations (Cyrkot et al., 2024), resulting in more decisions that breach moral norms.

Alternatively, paranoia leads to a suspicious attitude toward others, marked by anger and impulsivity, which significantly predicts later violent behavior (Appelbaum et al., 2000), leading to judgments that disregard moral norms. Similarly, negative rumination can exacerbate depressive symptoms, leading to increased hostility and fewer deontological decisions. Depressed individuals exhibit heightened levels of hostility (Moreno et al., 1993). Such hostile emotions may produce higher levels of moral disengagement (Shen et al., 2019), decrease personal moral responsibility (De Caroli and Sagone, 2014), and promote emotional and cognitive responses that lead to aggression (Bandura et al., 1996; Obermann, 2011). Consequently, this reduces deontological tendencies and leads to more decisions that violate moral norms.

According to the dual-process theory (Greene, 2009), utilitarian judgments may emerge from the efficient cognitive regulation of negative emotions. This framework posits that disruptions in baseline mood, such as those found in mood disorders, could lead to a rejection of utilitarian choices that require emotionally challenging decisions. Mood-altering states, including those induced pharmacologically, may amplify negative attitudes toward utilitarian judgments (Ngo et al., 2019). Therefore, depression may lead to increased deontological tendencies or decreased utilitarian tendencies. However, our study finds that depression reduces deontological tendencies as well as utilitarian tendencies. This is consistent with Moll and de Oliveira-Souza’s (2007) view that emotional and cognitive processes are not only concurrent but may also compete, influencing moral choices in different directions depending on the context and the health of these neural systems. This competition is apparent in frontal brain areas such as the VMPFC and the DLPFC, responsible for integrating emotional and cognitive evaluations (Moll and de Oliveira-Souza, 2007).

Our findings reveal that both tendencies were simultaneously reduced in adolescents with clinical depression. Traditional dual-process models generally suggest that deontology and utilitarianism are conflicting. Emotion, intuition, and automatic processing drive deontological reasoning, while deliberate cognitive processing drives utilitarian reasoning (Greene, 2009). However, our findings indicate that these two moral orientations are not mutually exclusive; they can both increase or decrease simultaneously. This aligns with the perspective proposed by Conway and Gawronski (2013), which argues that deontology and utilitarianism are not conflicting moral theories, but can coexist within the moral reasoning process. Our research extends the dual-process theory and supports the view that deontological and utilitarian reasoning are independent processes.

In our study, we adopted the Process Dissociation (PD) procedure, which offers advantages over the traditional moral dilemma paradigm. Unlike the conventional view that treats deontology and utilitarianism as opposing ends of a single continuum—where an increase in one implies a decrease in the other—the PD procedure conceptualizes them as independent cognitive processes (Conway and Gawronski, 2013). This distinction is crucial, as individuals can exhibit high or low levels of both deontological and utilitarian tendencies simultaneously. The PD procedure allows for the separate estimation of these two moral inclinations at the individual level. Compared to more recent paradigm, the CNI model (Gawronski et al., 2017) and the CAN algorithm (Liu and Liao, 2020), the PD procedure also offers greater statistical flexibility. It is well-suited for a range of complex analyses, including *t*-tests, ANOVA, regression models, and particularly the chain mediation analysis employed in our study. Therefore, the PD procedure was chosen not only for its conceptual strengths in distinguishing between independent moral processes, but also for its practical advantages in facilitating rigorous statistical analysis.

While we maintain that the PD procedure remains a valuable tool for investigating moral judgments, its application to moral dilemmas is not without limitations. Although the PD procedure distinguishes conceptually and empirically between utilitarian and deontological inclinations, it inadvertently conflates these inclinations with a general preference for action versus inaction. Specifically, the U parameter integrates both sensitivity to consequences and a tendency toward action, whereas the D parameter combines sensitivity to moral norms with a predisposition toward inaction. In response to these shortcomings, scholars have introduced alternative models—such as the CNI model (Gawronski et al., 2017) and the CAN algorithm (Liu and Liao, 2020)—which aim to more accurately capture the cognitive processes underlying moral judgments. Future research might consider using the CAN and CNI models to further validate the key findings of the current study.

The present study has several limitations. First, although the sample in Study 1 met the criteria established by the G*Power analysis, and the analyses of study 1 controlled for age-related variance, the relatively wide age range of adolescents (12–20 years) may require larger samples to examine effects in participants with different age. Future research should consider expanding the sample to further validate the findings of this study. Also, the significant age difference between the depression group (*M* = 16.97) and control group (*M* = 15.24) is concerning given the rapid development of moral reasoning during adolescence, and future research should more rigorously control for age matching between the clinical depression group and the control group.

Second, self-report measures for depression were used in the current research. Although self-report measures for depression are widely used in psychological research due to their convenience and efficiency, it still has some limitations. Participants may respond in ways that they believe are socially acceptable or expected. Also, self-report measures rely on the individual’s own perception of their emotions and symptoms, which can be influenced by their current mood, level of self-awareness, or ability to articulate their experiences. This introduces variability that might not accurately reflect their true psychological state. Meanwhile, some individuals, especially those with depression, may lack insight into their own condition, leading to underreporting or inaccurate reporting of symptoms. Future studies could benefit from incorporating assessments from doctors, and behavioral or neurophysiological assessments.

Third, the cross-sectional design precludes causal inferences; longitudinal or experimental designs are needed to disentangle temporal relationships between depression and moral judgments. Also, regarding mediating mechanisms, future studies should further explore the roles of diverse emotion regulation strategies, such as acceptance, attentional deployment, and reappraisal. Identifying additional mediators beyond hostility and paranoid ideation—such as cognitive flexibility, reasoning biases, or the cognitive disengagement/apathy discussed earlier—is also critical. Integrating both cognitive and emotional factors could provide a more holistic understanding of moral judgment mechanisms in depression.

Finally, our sample was restricted to adolescents in one city in China. Future research could validate this finding in a broader range of regions. Also, cultural factors may influence the relationship between depressive symptoms and decision-making patterns, as evidenced by racial differences in family decision-making dynamics (e.g., greater adolescent autonomy correlates with fewer depressive symptoms in White families but more in Black families (Schweingruber and Kalil, 2000). Cross-cultural replications are warranted to assess the universality of our findings.

## 5 Conclusion

This research has systematically explored the impact of both clinical depression and subclinical depressive symptoms on moral judgments among adolescents. The findings demonstrate that clinical depression significantly influences moral reasoning, reducing reliance on both utilitarian and deontological principles. Whereas, subclinical depression is negatively associated with deontological tendencies in moral judgments. Negative rumination can worsen subclinical depressive symptoms and then lead to higher paranoid ideation/hostility, consequently decreasing deontological tendencies, i.e., the sequential mediations of “negative rumination → depression → paranoid ideation → deontological tendency” and “negative rumination → depression → hostility → deontological tendency.” These findings underscore the importance of addressing depressive symptoms to better understand their broader implications on moral development among adolescents.

## Data Availability

The raw data supporting the conclusions of this article will be made available by the authors, without undue reservation.

## Appendix

### Crying Baby

#### Incongruent dilemma

It is war time. Enemy soldiers have taken over your village. They have orders to kill all remaining civilians. You and some of your townspeople have sought refuge in the cellar of a large house. Outside you hear the voices of soldiers who have come to search the house for valuables. A baby with no parents begins to cry loudly. You cover her mouth to block the sound. If you remove your hand from the baby’s mouth her crying will summon the attention of the soldiers who will kill you and the others hiding out in the cellar. To save yourself and the others you must smother the child to death. Is it appropriate for you to smother the child in order to save yourself and the other townspeople from being killed?

#### Congruent dilemma

It is war time. Enemy soldiers have taken over your village. They have orders to capture all remaining civilians to make them work quarrying stone in a mine. You and some of your townspeople have sought refuge in the cellar of a large house. Outside you hear the voices of soldiers who have come to search the house for valuables. A baby with no parents begins to cry loudly. You cover her mouth to block the sound. If you remove your hand from her mouth the crying will summon the attention of the soldiers who will capture you and the others hiding out in the cellar. To save yourself and the others from laboring in the mine you must smother the child to death. Is it appropriate for you to smother the child in order to save yourself and the other townspeople from being captured?

### Vaccine policy

#### Incongruent dilemma

You are a doctor in a health clinic overrun by patients with a serious disease. You just received a shipment of drugs that can cure the disease but the drugs have their own severe side-effects. If you administer the drugs to your patients, a small number will die from the side effects but most will live. If you do not, most will die from the disease. Is it appropriate for you to administer the drug to your patients?

#### Congruent dilemma

You are a doctor in a health clinic overrun by patients with the latest flu virus. You just received a shipment of drugs that can cure the flu but the drugs have their own severe side-effects. If you administer the drugs to your patients, a small number will die from the side effects but most will live. If you do not, most will continue to suffer from the effects of the flu virus for some time. Is it appropriate for you to administer the drug to your patients?
